# Anticoagulation Knowledge Tool (AKT): Further evidence of validity in the Italian population

**DOI:** 10.1371/journal.pone.0201476

**Published:** 2018-08-14

**Authors:** Arianna Magon, Cristina Arrigoni, Tiziana Roveda, Paola Grimoldi, Federica Dellafiore, Marco Moia, Kehinde O. Obamiro, Rosario Caruso

**Affiliations:** 1 Health Professions Research and Development Unit, IRCCS Policlinic San Donato University Hospital, San Donato Milanese, Milan, Italy; 2 Department of Public Health, Experimental and Forensic Medicine, Section of Hygiene, University of Pavia, Pavia, Italy; 3 Haemostasis and Thrombosis Center, Cardiological Department of ASST Sette Laghi, Galmarini Hospital, Tradate, Varese, Italy; 4 Angelo Bianchi Bonomi Hemophilia and Thrombosis Center, Department of Clinical Sciences and Community Health, University of Milan, IRCCS Ca’ Granda Maggiore Hospital Foundation, Milan, Italy; 5 Centre for Rural Health, College of Health and Medicine, University of Tasmania, Tasmania, Australia; Universite de Bretagne Occidentale, FRANCE

## Abstract

**Introduction:**

Oral Anticoagulation therapy (OAC) is highly effective in the management of thromboembolic disorders. An adequate level of knowledge is important for self-management and optimizing clinical outcomes. The Anticoagulation Knowledge Tool (AKT) was developed to assess OAC knowledge and caters for both patients prescribed direct oral anticoagulants or vitamin K antagonist (VKA). However, evidence regarding its psychometric proprieties, validity and reliability are unavailable in non-English speaking settings. For this reason, the aim of this study is to provide further evidence of validity for AKT and also developing an Italian AKT version (I-AKT) supported by evidence of validity and reliability.

**Methods:**

A multiphase study was conducted which included the following: cultural and linguistic validity; i.e. content validity; construct validity; reliability assessment. The Construct validity was performed using the contrasted group approach using three groups comprised of health care providers, patients and the general public. Furthermore, Exploratory Structural Equation Modelling (ESEM) was performed to confirm the mono-dimensional structure of the items in the AKT.

**Results:**

In construct validity phase 334 participants were enrolled. One-way ANOVA and post hoc analysis test demonstrated significant differences between the means knowledge scores of the three groups: 30.42±3.04 *vs* 23.45± 4.57 *vs*14.32±6.07 (Statistic F = 266.83; p < .001). ESEM analysis demonstrates the I-AKT mono-dimensionally structure with an explained variance of 56.42%. The scale also showed both good internal consistency reliability (Cronbach’s α = 0.896) and test-retest reliability (r = 0.855).

**Conclusion:**

This study developed and validated I-AKT with supporting evidence for validity and reliability. The study also confirms the mono-dimensional of the items in the AKT. This suggest that the instrument can be useful in non-English setting for knowledge assessment and in potentially developing patient education materials.

## Introduction

Oral anticoagulant (OAC) therapy is indicated in the management of non-valvular atrial fibrillation (NVAF) in order to prevent incidence of ischemic stroke, as well as in other medical conditions that are associated with thromboembolic complications [1,2]. OACs are broadly classified into two groups; the vitamin K antagonists (VKAs) and the direct oral anticoagulants (DOACs) [2]. The VKAs (e.g., warfarin, acenocoumarol) have been in use for more than 50 years and require intensive coagulation monitoring [3]. Additionally the VKAs are characterized by wide variation in dose-response relationships, and multiple drug-food and drug-drug interactions [3]. The DOACs (dabigatran, apixaban, rivaroxaban and edoxaban) have only been recently introduced into clinical practice, and are expected to overcome some of the limitations of warfarin therapy [3]. As per Italy, recent estimation studies suggested that nearly one million people are currently prescribed OAC medications [1].

OACs are high-risk medication and complex patient understanding is required to promote safe and effective use of OAC [4]. Patients who are well aware of the risk and benefit of OAC therapy are more likely to have better adherence compared to those who do not [5]. Furthermore, patients’ knowledge of their condition and OAC therapy have been reported to positively impact on anticoagulation control [6,7].

Due to the importance of OAC knowledge, a number of tools have developed to assess patients’ knowledge. Many of these tools however have been limited due to absence of information regarding their validity and reliability. In cases where validity and reliability analyses have been conducted, the instruments developed were not designed to assess knowledge regarding the recently introduced DOACs [8]. To overcome this limitation, the Anticoagulation Knowledge Tool (AKT) was recently developed [9]. The Anticoagulation Knowledge Tool (AKT) has been recently developed with supporting evidence for validity and reliability to fill this gap.

However, a limitation to the use of the AKT is the absence of evidence supporting validity and reliability in non-English speaking population, such as the Italian population [10], and the need to further explore its psychometric proprieties using an underlying variable approach [11]. This would be useful in confirming that the AKT adequately measures OAC knowledge and can be useful in other population, giving more possibility to compare results when the tool will be used in different language versions. Therefore, the purpose of this study is to provide further evidence of validity for AKT, describing its psychometric proprieties, and finally to develop an Italian version of the AKT (I-AKT).

## Materials and methods

In order to achieve the above purposes, a multiphase study described below was conducted.

### AKT description

AKT is a self-report tool, with evidence of construct validity and reliability (i.e., stability and internal consistency) [9]. It is composed by 28 items, with open-ended and multiple choice questions, divided into two sections: the first section (n = 20 items) assesses the general anticoagulation knowledge for all the available OAC, while the second section (n = 8 items) is specific to VKAs therapy. To each given answer, a zero (incorrect answer) or a one score (correct answer) is attributed by the clinicians to assess the patient knowledge. A maximum score of 25 is achievable for the first section, and a maximum score of 35 for patients taking VKAs who are required to complete both sections. Construct validity was assessed using the contrasted group approach, by comparing the knowledge scores of three groups of participants hypothesised to have different level of OAC knowledge. The three groups are: healthcare providers, patients receiving OAC treatments, and participants from the general public [9].

### Phase one: Cultural and linguistic validation

This phase aimed to provide a clear and culturally acceptable translation of AKT items in Italian language (I-AKT). As such, this phase was conducted according using the Brislin’s translation model for cross-cultural research, which involves performing a translation, back-translation and forward translation [12]. The first step involved two bilingual experts (AM, RC) who independently provided a draft version of AKT translation from English to Italian. Subsequently, the preliminary I-AKT version was shared in a consensus discussion group, involving anticoagulation experts and one patient, all with a good knowledge of the English language (A2 or B1 level), to define the most representative and comprehensible linguistic form for each translated item. The translated items were then assessed using a four point Likert scale score (1 = completely disagree; 4 = completely agree). Furthermore, a second translation from Italian to English was repeated by two additional bilingual investigators (RC, FD). The aim was to establish that the previously Italian-translations had retained their original meaning (i.e., back translation). Lastly, to further ensure the accuracy of the original translation, two other bilingual investigators (PG, TR), translated the AKT item from English to Italian (i.e., forward translation). Subsequently, the final version of the I-AKT was tested in a group of 10 adult volunteer patients, who have been receiving OAC therapy for at least three months and with good cognitive abilities, assessed using six-item screener test [13]. This pilot testing was performed to assess the clarity of the items using a four point Likert scale (1 = not clear, 4 = completely clear). The degree of agreement in the discussion group and among patients involved in pilot testing was assessed using Fleiss’ Kappa index. A value of 0.70 was considered as the cut-off point to indicate adequate consensus [14].

### Phase two: Quantitative and qualitative content validity

We proceeded by testing the I-AKT version for both quantitative and qualitative content validity, following standardized methodology. The quantitative content validity was conducted according to the approach described by Lawshe [15]. This phase involved a group of 14 anticoagulation experts with the aim of rating the pertinence (i.e., essential contents) and the relevance (i.e., appropriateness) of each item in regards to the theoretical construct given by the OAC knowledge. For this reason, two specific index of quantitative content validity were computed to assess the level of agreement among the raters: (a) Content Validity Ratio (CVR) to assess the pertinence through a three-point ordinal scale (1 = not pertinent; 2 = useful but not pertinent; 3 = highly pertinent) and (b) Content Validity Index (CVI) to assess relevance, through a four-point ordinal scale (1 = not relevant; 2 = somewhat relevant; 3 = quite relevant; 4 = highly relevant) [16]. Specifically, each item was considered pertinent when the obtained CVR matched a score ≥ .65, computed with the following formula CVR = (N*e*-N/2)/(N/2), in which the N*e* represented the number of rates indicating ‘pertinence = 3’ and N the total number of total rates [15]. According to the formula described by Lawshe. The CVI index was calculated using two approaches. Firstly, we computed item level (I-CVIs), followed by scale level (S-CVI) using the average of the I-CVIs scores as described by Polit [16].

Using the same panel of experts, we assessed the qualitative content validity index (i.e., face validity) to understand the clarity and comprehensibility of the items and discuss any potential ambiguity. This was evaluated using three open questions, and responses were analyzed using a narrative analysis method to summarize the main emerging themes (i.e., textual content analysis) [17].

### Phase three: Construct validity (psychometric proprieties assessment)

This phase was performed using a cross-sectional study design, and a convenience sampling. According to the approach used to develop the original AKT (i.e., contrasted group approach), we involved three different groups of respondents with hypothesized different OAC knowledge levels: healthcare providers (group 1), patients receiving OAC treatment (group 2), and general public (group 3). Healthcare providers were eligible to participate if they have greater than five years’ experience in managing patients taking OAC. The inclusion criteria for patients receiving OAC treatment were: (i) age ≥ 18 years, (ii) managed by an anticoagulation clinic for at least three months, (iii) adequate cognition evaluated using the six-item screener [18], and (iv) able to provide consent to be enrolled in the study. The exclusion criteria for participants in this group was a high Charlson Comorbidity Index (CCI) (CCI > 4), to control for potential variability as a result of comorbidity and to ensure homogeneity of the study sample [19]. For the general public group, the inclusion criteria were: (a) age ≥ 18 years, (b) have never received OAC therapy. Furthermore, participants were excluded from the general public group if they have any of the following: (a) close relation or friend receiving OAC treatment, (b) CCI > 4, (c) cognitive impairment.

Data were collected from February to July 2017. Participants in the healthcare provider and patient groups were enrolled by two different anticoagulation clinics in North Italy. Eligible healthcare providers (group 1) and patients under OAC treatment (group 2) were identified by the medical facilities. Participants from the general public (group 3) were enrolled by the research team. Participants across the groups were enrolled after they had received detailed information regarding the study and have given written informed consent. Participants in groups 1 and 3 were asked to assume they were receiving OAC treatment while completing the questionnaire. Group 2 participants were asked to complete the I-AKT based on their diagnosed clinical condition. Demographic information including, gender, age in years, marital status, nationality, education, employment were collected from all participants. In addition, participants in group 2 were asked to provide information related to their condition and OAC therapy including, the name of prescribed OAC, clinical indication, thromboembolic or bleeding complications in the last three months, duration of therapy, quality of anticoagulation control (i.e., time in therapeutic range, TTR). Lastly, a second test was conducted for 10 randomly selected participants from each group after a period of 30 days.

### Statistical analysis

Descriptive statistics was used to describe the sample characteristics. Categorical data were presented as frequencies, while continuous data were presented as means ± standard deviation (SD) for normally distributed variables, and as median and interquartile range (IQR) non-normally distributed data. Missing data were managed using a pairwise approach.

The construct validity was assessed using both the contrasted group approach [20], and an underlying variable approach for dichotomous variables, using Exploratory Structural Equation Modelling (ESEM) [21]. The contrasted group approach tested the hypothesis of significant differences in mean scores between the three groups. One-way analysis of variance (ANOVA) with Tukey’s post-hoc analysis was used to detect the differences between groups. ESEM provides more parameters than the classical exploratory factorial analysis to interpret the model, as well as the structural equation modelling. ESEM was performed on the patient group (group 2) because it represented the real OAC patients. The sampling adequacy was tested using Kaiser-Meyer-Olkin (KMO) test and the obtained tetrachoric correlation matrix was tested for factorability using the Bartlett’s test of sphericity. ESEM was performed to test the theoretical mono-dimensional structure hypothesis (i.e., AKT measures the knowledge domain) using Weighted Least Square method (WLS-MV) estimator of MPlus, which is the suitable estimator for scales having ordinal or dichotomous variables [21]. ESEM models were evaluated using the following criteria: adherence to theoretical structure (mono-dimensional as the most plausible model), factor loadings for standardized solutions ≥ .40, interpretation of eigenvalues scree test, interpretation of Goodness of Fit Statistics (fit indices), and the model explained variance. Those fit indices were considered: *χ*2 (omnibus test) where p should be significant, the Incremental Fit Indices (CFI, value should be ≥ 0.90), the Root Mean Square Error of Approximation (RMSEA, value should be < 0.06), and the Standardized Root Mean Square Residual (SRMR, value should be ≤ 0.08). The I-AKT internal consistency reliability was assessed using Cronbach’s α, and by the stability. Randomization sequence to select participants for re-test was created using Excel 2007 (Microsoft, Redmond, WA, USA) and assessed using Pearson product-moment correlation coefficient (r). All data were analyzed using Statistical Package for Social Science version 22 (SPSS, Chicago, IL, USA) and MPlus 8.1. The level of significance of each test was set at 0.05 and two-tailed.

### Ethical considerations

This study was approved from the Research & Ethical Committee of San Raffaele Hospital (Italy) (Protocol n. 84/int/2017 of 27^th^ January 2017) ensuring the data collection and its’ management in accordance with international ethical principles (Good Clinical Practice, GCP) and Italian legal and research ethics requirements for non-interventional studies. This study was conducted using a multicenter design, involving two main anticoagulation clinics in North Italy, and all the participants were informed about the aims and the method of the study, and they were asked to provide written informed consent, as required in the Italian Legislative Decree 196 of 30^th^ June 2003. Participants of each phase were also informed about the confidentiality of their responses and anonymity in the presentation of data for the final report of the study.

## Results

### Phase one: Cultural and linguistic validation

Agreement on the final I-AKT draft was reached by the consensus discussion group which involved nine healthcare providers and one patient (n = 4 nurses; n = 4 physicians; n = 1 pharmacist; n = 1 patient). The participants were 70% women, with a median age of 40.5 years (IQR = 18.3 years), and 80% of them had university level education. The level of agreement on the comprehensibility of each Italian translated item computed through the Fleiss’ K index was equal to 0.84. For the pilot test conducted, the level of agreement among patients taking OAC, also computed through the Fleiss’ K index was 0.90.

### Phase two: Quantitative and qualitative content validity

The panelist group involved in the evaluation of the relevance and pertinence of each item of the I-AKT was performed by 14 anticoagulation experts (n = 3 physicians; n = 10 nurses; n = 1 pharmacist). It was composed by 78.6% of females, with median age of 33 years (IQR = 13.5 years), and all experts presented a university level of education. All the computed CVRs were higher than 0.86. The I-CVIs were higher than 0.93, with a final S-CVI of 0.99 (Table 1). Finally, textual analysis showed the items of the I-AKT to be clear and useful, without requiring any further modification.

**Table 1:** CVR, I-CVIs, S-CVI.

Panelists (n = 14)					
	CVR	Interpretation	I-CVI	Interpretation	S-CVI
**SECTION A**					
**Item 1** What is the name of your anticoagulation medicine? *(Qual'è il nome del farmaco anticoagulante che Lei assume*?*)*	1.00	Essential	1.00	Relevant	0.99
**Item 2** Why has your doctor prescribed you this medicine? *(Per quale motivo il Suo medico Le ha prescritto questo farmaco*?*)*	1.00	Essential	1.00	Relevant	
**Item 3** How does this medicine work in your body? *(Come funziona questo farmaco*?*)*	1.00	Essential	1.00	Relevant	
**Item 4** How many times a day do you need to take this medicine? *(Quante volte al giorno è necessario che Lei assuma questo farmaco*?*)*	1.00	Essential	1,00	Relevant	
**Item 5** For how long do you need to take this medicine (for example, 3 months, and 6 months, life-long)? *(Per quanto tempo è necessario che Lei assuma questo farmaco (ad esempio*, *per tre mesi*, *per sei mesi*, *o per tutta la vita)*?	1.00	Essential	1.00	Relevant	
**Item 6** Why is it important to take this medicine exactly as your doctor has told you? *(Per quale motivo è importante che Lei assuma questo farmaco esattamente come le ha detto il Suo medico*?*)*	0.86	Essential	0.93	Relevant	
**Item 7** Is it important to take this medicine at the same time each day? *(E’ importante assumere questo farmaco alla stessa ora*, *ogni giorno*?*)*	1.00	Essential	1.00	Relevant	
**Item 8** Is it okay to double the next dose of this medicine if you miss a dose? *(E’ corretto raddoppiare la dose successiva in caso di mancata assunzione di una dose di questo farmaco*?*)*	1.00	Essential	1.00	Relevant	
**Item 9** Is it possible that skipping one dose of this medicine could worsen your condition? *(Saltare l’assunzione di una dose di questo farmaco può peggiore il suo stato di salute*?*)*	1.00	Essential	1.00	Relevant	
**Item 10** Is it appropriate to stop taking this medicine once you feel better? *(E’ giusto interrompere l’assunzione di questo farmaco una volta che Lei si sete meglio*?*)*	1.00	Essential	1.00	Relevant	
**Item 11** Is safe to take anti-inflammatory medicines like ibuprofen (Nurofen^®^ or Advil^®^) while you are taking this medicine? *(E’ sicuro assumere farmaci anti-infiammatori*, *ad esempio Ibuprofene (Brufen*^®^*) mentre è in terapia con questo farmaco*?*)*	1.00	Essential	1.00	Relevant	
**Item 12** Is it safe to take vitamin supplements and herbal medicines with this medicine without consulting your doctor? *(E’ sicuro assumere integratori vitaminici o prodotti di erboristeria durante l’assunzione di questo farmaco senza consultare il Suo medico*?*)*	1.00	Essential	1.00	Relevant	
**Item 13** Is there any benefit in taking more of this medicine than your doctor has told you take? *(C'è qualche beneficio nell’aumentare il dosaggio di questo farmaco rispetto a quello che Le ha detto il Suo medico di assumere*?*)*	0.86	Essential	0.93	Relevant	
**Item 14** Will drinking too much alcohol increase the risk of side effects with this medicine? *(Bere troppi alcolici aumenta il rischio di effetti collaterali durante l’assunzione di questo farmaco*?*)*	1.00	Essential	1.00	Relevant	
**Item 15** Would you inform a surgeon, dentist or other health professional that you are taking this medicine before undergoing surgery or a procedure? *(Informerebbe il chirurgo*, *il dentista o altri professionisti sanitari dell’assunzione di questo farmaco prima di sottoporsi a chirurgia o altre procedure*?*)*	1.00	Essential	1.00	Relevant	
**Item 16** Is it important that all the health care practitioners you see know that you are taking this medicine? *(E’ importante che tutti i professionisti sanitari che la seguono sappiano che Lei sta assumendo questo farmaco*?*)*	1.00	Essential	1.00	Relevant	
**Item 17** What is the most important side effect of this medicine? *(Qual è il più importante effetto collaterale di questo farmaco*?*)*	1.00	Essential	1.00	Relevant	
**Item 18** THREE signs of side effects that you should watch out for while taking this medicine are: *(Elenchi tre segni di effetti collaterali ai quali dovrebbe fare attenzione durante l’assunzione di questo farmaco)*	0.86	Essential	0.93	Relevant	
**Item 19** THREE things you can do to reduce your risk of side effects are *(Elenchi TRE cose che Lei può fare per ridurre il rischio di eventi collaterali)*	0.86	Essential	0.93	Relevant	
**Item 20** What is the best step to take if you accidentally take too much of this medicine? *(Qual'è la migliore cosa che Lei può fare nel caso in cui accidentalmente avesse assunto una quantità eccessiva di questo farmaco*?*)*	0.86	Essential	0.93	Relevant	
**SECTION B**					
**Item 1** What is your target INR range? *(Quale deve essere il Suo intervallo di INR*?*)*	1.00	Essential	1.00	Relevant	
**Item 2** What was your last INR reading? (*Quale era il Suo ultimo valore di INR*?*)*	1.00	Essential	1.00	Relevant	
**Item 3** Are regular INR tests necessary to know how well this medicine is working? (*Sono necessari controlli regolari di INR per sapere se il farmaco che sta assumendo funziona*?*)*	1.00	Essential	1.00	Relevant	
**Item 4** Is an INR value above your target range good for general wellbeing? *(Un valore di INR al di sopra dell’intervallo di riferimento è positivo per il suo stato di salute*?*)*	1.00	Essential	1.00	Relevant	
**Item 5** Is it possible for INR values below your target range to be bad for your health? (*Un valore di INR al di sotto dell’intervallo di riferimento è negativo per il Suo stato di salute*?*)*	1.00	Essential	1.00	Relevant	
**Item 6** Is it possible for what you eat to affect your warfarin therapy? *(Quello che Lei mangia può influenzare l’efficacia di questo farmaco*?*)*	1.00	Essential	1.00	Relevant	
**Item 7** If you answered ‘Yes’ above, list THREE foods that can affect your anticoagulant therapy *(Se ha risposto Sì alla domanda precedente*, *elenchi TRE alimenti che possono influenzare la terapia anticoagulante)*	1.00	Essential	1.00	Relevant	
**Item 8** List one vitamin that can significantly affect your anticoagulant therapy *(Elenchi una vitamina che può influenzare in modo importante la terapia anticoagulante)*	1.00	Essential	1.00	Relevant	

Note: original language was English. Italian version in italics.

### Phase three: Construct validity

#### Sample characteristics (n = 334)

**Fig 1:**
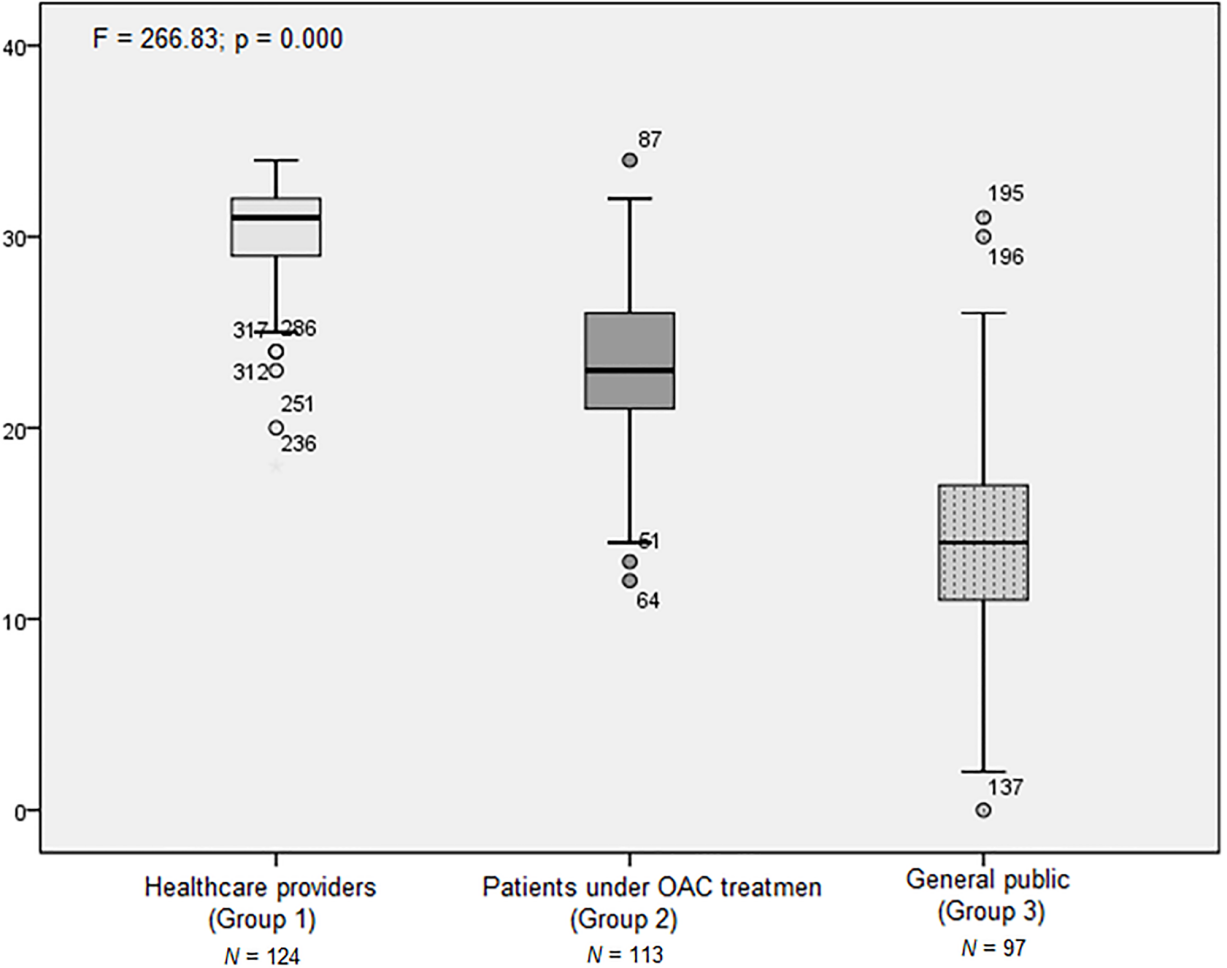
Mean scores comparison between the 3 groups. Tukey’s post-hoc test showed significant differences (p < .000) in comparing the means of all the groups (Group 1 vs Group 2; Group 1 vs Group 3; Group 2 vs Group 3).

Participants characteristics were described in Table 2. The contrasted group approach showed differences in all the comparisons (Fig 1). Specifically, the means ± SD of group 1, group 2, group 3 were 30.42 (±3.04), 23.45 (±4.57), 14.32 (±6.07), respectively (Statistic F = 266.83; p < 0.001). Tukey’s post-hoc test showed significant differences in all the comparisons, (each p was lower than 0.001).

**Table 2:** Demographic and clinical characteristics (phase 3) (n = 334).

		Group 1. Healthcare Providers (n = 124)	Group 2. Patients under OAC treatment (n = 113)	Group 3. General public (n = 97)
		n (%)	n (%)	n (%)
**Gender**				
	Male	38 (30.6)	72 (63.7)	49 (50.5)
	Female	86 (69.4)	41 (36.3)	48 (49.5)
**Marital Status**				
	Married	63 (50.8)	90 (79.6)	59 (60.8)
	Unmarried	61 (49.2)	23 (20.4)	38 (39.2)
**Nationality**				
	Italian	124 (100.0)	109 (96.5)	97 (100.0)
	Foreigners	NA	4 (3.5)	NA
**Education**				
	Primary school	NA	44 (38.9)^a^	20 (20.6)
	High school	27 (21.8)	58 (51.3)^a^	61 (62.9)
	Bachelor Degree	74 (59.7)	11 (9.7)^a^	16 (16.5)
	Post graduate	23 (18.5)	NA	NA
**Employment**				
	Employed	124 (100.00)*	17 (15.0)	66 (68.0)
	Not employed	NA	16 (14.2)	12 (12.4)
	Retired	NA	80 (70.8)	19 (19.6)
**Type of anticoagulation treatment**				
	DOAC	NA	8 (7.1)	NA
	VKA	NA	105 (92.9)	NA
**Clinical indication for anticoagulation treatment**				
	VTE	NA	17 (15.0)^a^	NA
	AF	NA	70 (61.9)^a^	NA
	Heart Valve Prothesis	NA	24 (21.2)^a^	NA
	PTA	NA	1 (0.9)^a^	NA
**Thromboembolic or Bleeding complications** §				
	Yes	NA	6 (5.3)	NA
	Not	NA	107 (94.6)	NA
**Time in treatment**				
	≥ 3 months	NA	4 (3.5)	NA
	≥ 6 months	NA	9 (7.9)	NA
	≥ 12 months	NA	22 (19.4)	NA
	≥ 36 months	NA	78 (69.0)	NA
**Quality of anticoagulation control**				
	TTR° 65–70%	NA	45 (39.8)	NA
**CCI**				
	No comorbidities	NA	2 (1.7)	69 (73.4)
	1–2		90 (79.6)	23 (24.4)
	3–4		21 (18.5)	2 (2.1)
		**Mean (SD)**	**Mean (SD)**	**Mean (SD)**
**Age** (years)		37.79 (10.63)	71.66 (9.86)	50.44 (17.57)

NA: Not applicable

* Health professionals: (a) Nurses (n = 118; 95.2%); (b) Physician (n = 5; 4.0%); Pharmacist (n = 1; 0.8%)

DOAC: Direct Acting Oral Anticoagulant

VKA: Vitamin K Antagonist

VTE: Venous Thromboembolism; this group also includes Ictus (n = 2; 1.8%) and Acute Myocardial Infarction (n = 2; 1.8%)

AF: Atrial Fibrillation (n = 36; 31.8%); Atrial Flutter (n = 2; 1.7%); Persistent atrial fibrillation (n = 32; 28.3%)

Heart Valve Prosthesis: includes, Mitral Valve Replacement (n = 8; 7.1%) and Aortic Valve Replacement (n = 16; 14.1%)

PTA: Percutaneous Transluminal Angioplasty

^§^ Thromboembolic or Bleeding complication in the last three months

° Time in Therapeutic Range (TTR) within 65–70% is a good standard of reference in the last three months

^a^ Percentage may not reach the 100% of the category representation, due to the pairwise management of the missing values

ESEM conducted to test mono-dimensionality showed adequate KMO, which was equal to 0.92, and adequate fit indices: *χ*2(324) = 751.825, p < 0.001; RMSEA = 0.059 (90%CI = 0.57–0.69), p < 0.001; CFI = 0.945; TLI = 0.940; SRMR = 0.04. The variance explained by this model was 56.42%. Factor loadings for this mono-dimensional solution are showed in Table 3.

**Table 3:** Factor loadings.

	Loadings
**Section A**	
Item1	0.862
Item2	0.746
Item3	0.634
Item4	0.827
Item5	0.630
Item6	0.629
Item7	0.577
Item8	0.670
Item9	0.466
Item10	0.633
Item11	0.484
Item12	0.572
Item13	0.664
Item14	0.460
Item15	0.676
Item16	0.803
Item17	0.742
Item18	0.775
Item19	0.770
Item20	0.539
**Section B**	
item1	0.927
item2	0.968
item3	0.770
item4	0.778
item5	0.651
item6	0.858
item7	0.920
item8	0.781

% explained variance is 56.42.

Estimates for factor loading derived from MPlus completely standardized solution.

Cronbach’s α was computed on the overall scale for group 2, and was equal to 0.896. Also test-retest reliability for participants across the three groups was showed a high and significant positive correlation (r = 0.855; p < 0.001).

## Discussion

Patients’ knowledge is a key element that can potentially affect adherence, and treatment outcomes in patients taking OACs [6,22,23]. For this reason, routine knowledge assessment is an important consideration in this group of patients. The AKT is a valid tool to objectively assess patients’ OAC knowledge by both healthcare providers and researchers. Healthcare providers can use AKT in their routine clinical practice to measure OAC knowledge in patients taking either VKAs or DOACs. Researchers can use AKT for research endeavors where it is necessary to assess OAC knowledge, such as in studies designed to describe the relations between OAC knowledge and health outcomes or to measure improvements in OAC knowledge when an educational intervention is tested. However, AKT was unavailable in Italian language, and therefore could not be utilized in the Italian healthcare setting. This study was designed to fill this gap by developing the I-AKT and provide further evidence on the validity of AKT.

Generally, the challenges related to the tools’ validity in cross-cultural research are mainly due to cultural-linguistic adaptation and content validity [12]. It is therefore important to follow clear methodological strategies to overcome those challenges, mainly arising from cultural and linguistic differences between the source and target languages. For this reason, the first two phases of this study focused on using best practices in ensuring linguistic and content validity of the I-AKT. These two phases gave a solid basis for the construct validity assessment (Phase 3).

I-AKT construct validity followed Terwee’s quality criteria recommendations [24], testing two main predefined hypotheses: (a) an expected differences in scores between ‘known’ groups, using the contrasted group approach [20], and (b) mono-dimensionality, using ESEM approach [21]. One-way ANOVA and Tukey’s post-hoc test confirmed that healthcare providers had better knowledge level than patients taking OAC, and patients’ knowledge was higher than that of participant from the general public. This suggests that the I-AKT scale is capable in discriminating between different knowledge levels. This comparison between groups has not to be intended for clinical implication, but only for a validity matter. Specifically, these results are in line with the theoretical expectation and with the findings of the original study [9]. Since the dimensionality of AKT has never been explored, this study contributes to existing knowledge using the ESEM analysis to provide further evidence of validity for this tool. The result suggests that the mono-dimensional structure of the AKT in assessing OAC knowledge is plausible: this result represents a novelty to further sustain AKT construct validity.

I-AKT reliability, assessed using internal consistency and test-retest reliability analysis, was also adequate. Specifically, internal consistency analysis showed that I-AKT items are interrelated, measuring the same construct, which is similar to the findings of the original study [9]. I-AKT had also good evidence of stability, considering that the correlation coefficient (r) between the two different measurements of test-retest was highly positive and significant. This implies that I-AKT can be useful to provide consistent score over time in a stable group of patients.

OAC knowledge in literature has often been assessed using a variety of methods devoid of clear validity. This has resulted in diverse observations. For example, some studies have showed a positive and significant correlation between patient knowledge and good anticoagulation control [7], while others have reported no significant correlation [25]. Our study which confirmed I-AKT construct validity using two different approaches suggests that this tool could potentially be useful in providing a clear relationship between OAC knowledge and other patient-related outcomes.

However, the present study is not without its potential limitations. The construct validity phase of the study employed a convenient sampling approach, except for participants who were re-tested to ascertain test-retest reliability. The following aspects have to be considered to correctly interpret the study results. Firstly, patients with CCI > 4 score were not included in the study. This implies that the evidence of validity and reliability is mainly applicable to patients with CCI score lower than four. Further, only 39.8% of patients taking OAC show a good quality of anticoagulation control, which is up to 60% in other Italian studies [1]. This may imply a possible selection bias, which is compatible with the convenience sampling or a need to a readdress the management strategy of Italian patients prescribed VKAs. However, the primary aim of this study is to validate the I-AKT and to provide psychometric analysis of the tool in the Italian setting. Another limitation is related to the small number of participants prescribed a DOAC (n = 8). This implies that the evidence of I-AKT validity is limited for this category of patients. Accordingly, we cannot affirm that our sample is fully representative of the Italian population taking OAC. This could affect the possible inferential analysis in assessing the relationships between study variables. For this reason, we chose not to perform analyses to explore the relationships between socio-demographic, clinical variables, knowledge, and anticoagulation control (TTR), due to the implicit risk of bias in the interpretation of the inferential analysis, considering the limits of our sample. However, this design is suitable for the study aim of developing and validating the I-AKT. The last limitation is related to the absence of criterion validity, using a measure of adherence (e.g. Morisky scoring) to determine the sub-groups stratification for the comparison and the testing of a priori framework, where patients with higher adherence should be those patients with higher knowledge. The main strengths of this study, however, is highlighted by the methodological approach in designing all the study phases, and by the ESEM analysis which provided new information about the validity and mono-dimensionality of the AKT.

## Conclusion

This study developed and validated I-AKT. Overall, the I-AKT showed good psychometric properties and appears to be potentially useful in clinical practice and research. Currently, it is limited to patients treated using VKAs and those with fewer co-morbidities. Future investigations required to establish the validity of the I-AKT in patients prescribed DOACs and those with CCI > 4. Also, further research is needed in understanding the relationships between knowledge and clinical outcomes like incidence of stroke and bleeding. We recommend the widespread use of AKT and I-AKT in assessing knowledge of patients taking OAC, due to evidence supporting their validity and reliability. In the Italian context, an epidemiological mapping of OAC patients’ knowledge will be useful in defining educational priorities and critically reflecting on the current overall OAC management strategy.

## Supporting information

S1 File Dataset I-AKT. (XLSX)
