# Correction: Anticoagulation Knowledge Tool (AKT): Further evidence of validity in the Italian population

**DOI:** 10.1371/journal.pone.0204534

**Published:** 2018-11-15

**Authors:** Arianna Magon, Cristina Arrigoni, Tiziana Roveda, Paola Grimoldi, Federica Dellafiore, Marco Moia, Kehinde O. Obamiro, Rosario Caruso

There is an error in affiliation 1 for authors Arianna Magon, Federica Dellafiore, and Rosario Caruso. Affiliation 1 should be: Health Professions Research and Development Unit, IRCCS Policlinico San Donato, San Donato Milanese, Milan, Italy.
